# Supplementary material for: A Systematic Review of Time Series Classification Techniques Used in Biomedical Applications
Source: Sensors (Basel). 2022 Oct 20;22(20):8016. doi: 10.3390/s22208016 (PMC9611376; doi:10.3390/s22208016)
Supplement: Supplementary file 1 [file sensors-22-08016-s001.zip › sensors-1893063-supplementary.pdf]

Supplementary Materials

Search terms

Supplementary Table S1. Search terms for each database.

Database	Search Term
PubMed	(“time-series*”[tiab] OR “time series*”[tiab] OR “temporal*”[tiab] OR “sequential*”[tiab] OR “continuous*”[tiab] OR “longitudinal*”[tiab]) AND (“classify*”[tiab] OR “classification*”[tiab] OR “algorithm*”[tiab] OR “machine learning*”[tiab]) AND (“*clinic*”[tiab] OR “physiology*”[tiab] OR “physiological*”[tiab] OR “biological*”[tiab] OR “*medical*”[tiab] OR “health*”[tiab] OR “sleep*”[tiab] or “activity*”[tiab]) NOT (“gene*”) NOT (“image*”[tiab] OR “imaging*”[tiab]) AND (“hidden markov model*”[tiab] OR “state space model*”[tiab] OR “state-space model*”[tiab] OR “wavelet transform*”[tiab] OR “time series classification*”[tiab] OR “time series classifier*”[tiab] OR “time series machine learning*”[tiab] OR “dynamic time warping*”[tiab] OR “DTW-kNN*”[tiab] OR “Time Warp Edit*”[tiab] OR “Move split merge*”[tiab] OR “derivative distance transform*”[tiab] OR “complex invariant distance*”[tiab] OR “bag of patterns*”[tiab] OR “bag-of-patterns*”[tiab] OR “Symbolic Aggregate Approximation - Vector Space Model*”[tiab] OR “SAXVSM*”[tiab] OR “Bag of SFA Symbols*”[tiab] OR “shapelet*”[tiab] OR “time series forest*”[tiab] OR “time series bag of features*”[tiab] OR “learned pattern similarity*”[tiab] OR “elastic ensemble*”[tiab] OR “collective of transformation ensembles*”[tiab] OR “rotation forest*”[tiab] OR “kalman filters*”[tiab] OR “ARMA model*”[tiab]) AND English[lang] NOT (“deep learning*”[tiab] OR “LSTM*”[tiab] OR “temporal CNN*”[tiab] OR “neural network”[tiab] OR “ResNet”[tiab] OR “LSTM”[tiab] OR “RNN”[tiab] OR “Long Short-Term Memory”[tiab])
IEEE	("Abstract": "time series") AND<br>("Abstract": "classification" OR "Abstract": "machine learning" OR "Abstract": "classifier" OR "Abstract": "algorithm") AND<br>("Abstract": "biomedical" OR "Abstract": "medical" OR "Abstract": "physiological" OR "Abstract": "biological") NOT<br>("Abstract": "deep learning" OR "Abstract": "lstm" OR "Abstract": "recurrent neural network" OR "Abstract": "neural network" OR "Abstract": "convolutional neural network" OR "Abstract": "rnn" OR "Abstract": "cnn")
Scopus	(TITLE-ABS-KEY("*time-series classification") OR TITLE-ABS-KEY("*time-series classifier")) AND (TITLE-ABS-KEY("*clinical") OR TITLE-ABS-KEY("*biomedical") OR TITLE-ABS-KEY("*physiological")) AND NOT (TITLE-ABS-KEY("*gene") OR TITLE-ABS-KEY("*imaging"))
Web of Science	KP =(("time series" OR time-series OR temporal) AND (classification OR "machine learning" OR classifier OR algorithm OR automatic OR “hidden markov model” OR “state space model” OR “state-space model*” OR “wavelet transform*” OR “time series classification*” OR “time series classifier*” OR “time series machine learning*” OR “dynamic time warping*” OR “DTW-kNN*” OR “Time Warp Edit*” OR “Move split merge*” OR “derivative distance transform” OR “complex invariant distance” OR “bag of patterns” OR “bag-of-patterns” OR “Symbolic

	Aggregate Approximation - Vector Space Model" OR "SAXVSM" OR "Bag of SFA Symbols" OR "shapelet" OR "time series forest" OR "time series bag of features" OR "learned pattern similarity" OR "elastic ensemble" OR "collective of transformation ensembles" OR "rotation forest" OR "kalman filters" OR "ARMA model") NOT ("RNN" OR "deep learning*" OR "LSTM" OR "temporal CNN*" OR "Neural Network*")) AND<br>TS= (biomedical OR medical OR physiological OR biological NOT gene* NOT image*)

Algorithm Types and Example Papers

Supplementary Table S2. Description for each algorithm type and example papers.

Best Algorithm Type	Description	Example papers
Feature extraction	The type of time series classification technique where features are extracted to describe a particular time series sample and the features are fed into traditional machine learning algorithms as inputs of the predictive modeling.	See Supplementary Table S4a,b
Distance-based methods	This method is based on defining or quantifying the difference between 2 or more time series data samples. The first class of classification methods rely on some notion of distance between two time series or time series subsequences. Intuitively, a distance can be seen as a proxy for dissimilarity. Two time series that are in close proximity (i.e., they have a small distance) under some distance measure, are likely to come from the same class.	[79]
Shapelet based	Shapelet-based methods are similar to significant pattern mining. Time series shapelets are subsequences that maximize classification performance.	[1]
Statistical Modeling	This technique uses statistical modeling (such as Kalman filters or Hidden Markov models) to describe or fit to the time series observed. Using the information obtained from statistical models, we can make decisions or extract features to be used as inputs to machine learning algorithms.	[37]
Wavelet Transform	Wavelet Transform can be used for signal cleaning (preprocessing), signal decomposition (preprocessing) as well as feature extraction. This technique is widely used and can be considered an integral part of time series machine learning.	[26]
Ensemble-based	Ensemble-based classification algorithms utilize multiple algorithms to make predictions and then aggregate the results coming from these different algorithms	[54]

Non-linear index and thresholding	This time series classification method is based on defining indices based on domain- and data-driven time series features. The thresholds for these indices can be predefined or found through statistical learning. The thresholds are then used to make predictions of classes.	[62]

Preprocessing Methods

Supplementary Table S3. Summary of time series signal preprocessing methods and example papers.

Preprocessing Steps	Preprocessing Method	Example Papers
Filtering or filters	• High Pass Filter<br>• Low Pass Filter<br>• Butterworth Filter<br>• Notch Filter<br>• Moving Average Filter<br>• Median Filter<br>• Wiener Filter<br>• Wavelet FilterSoftware<br>• Chebyshev Type II Band-pass Filter	[55,80,81]
Artifact removal	• Component Analysis<br>• Noise<br>○ Discrete Wavelet Transform<br>• Anonymous Amplitude<br>• Detrending<br>○ Locally Estimated Scatterplot Smoothing (LOESS)<br>• Outlier Detection	[33,70]
Segmentation	• Sliding Window<br>• Interval Proportion<br>• Data Augmentation<br>• Pan-Tompkins Algorithm<br>• Thresholding<br>• Linear interpolation of ectopic beats<br>• Window Discretization	[71,82]
Resampling	• Downsampling<br>• Downscaling	[41]
Smoothing	• Exponential Smoothing<br>○ Single<br>○ Double	[56,81]

	■ Fuzzy Inference Double Exponential Smoothing (FIDES)<br>○ Tripple<br>● Smoothing Filter<br>○ Mean Filter<br>○ Moving average filtering<br>○ Median filtering<br>○ Three point linear smoothing filtering<br>○ Five point linear smoothing filtering.	
Dimension reduction	● Autoregressive (AR) Model<br>● Principal Component Analysis (PCA)	[83]
Synthetic Data Generation using statistical explosion	● Data explosion model	[29]
Data cleaning	● Ensemble of various methods like<br>○ outlier detection<br>○ data balancing(undersampling)<br>○ selective removal of missing data.	[29,54]
Other	● Normalization<br>● Squared<br>● Multivariate feature discretization<br>● Sub-sequence Enumeration<br>● Data Transformation<br>● Data imputation	[24,84]

Feature Engineering Techniques and Signal Specific Feature Engineering

Supplementary Table S4. (a) Example of feature engineering techniques and papers. (b) Features for common signals and example papers.

**(a)**		
**Feature Type**	**Feature Names**	**Example Papers**
Amplitude	● Mean absolute value<br>● Arithmetic mean<br>● Harmonic mean<br>● Geometric mean<br>● Root mean square	[23,85]

	• Skewness<br>• Kurtosis<br>• Minimum<br>• Maximum<br>• Mode<br>• First Higher-Order Mean Absolute Value<br>• Normalized 1st Higher-Order Mean Absolute Value<br>• Second Higher-Order Mean Absolute Value<br>• Normalized 2nd Higher-Order Mean Absolute Value<br>• Peak-to-peak Amplitude<br>• Peak Amplitude<br>• Mean Relative Time of the Peaks<br>• Mean Relative Time of the Valleys	
Frequency	• Trend<br>• Seasonality<br>• Total power in frequency band<br>• Power spectral density<br>• Bandwidth<br>• Center Frequency<br>• Median Frequency<br>• Mean Frequency<br>• Mode Frequency<br>• Root Mean Square Successive Interval Differences<br>• Ratio between Low Frequency and High Frequency (LF/HF)<br>• Mean normal-to-normal interval (NNI)<br>• Standard deviation of normal-to-normal interval (SDNNI)<br>• Root mean square of successive differences between NNIs (RMSSD)<br>• Number of NNIs > 20 ms (NN20)<br>• Proportion of NN20 among total number of NNIs (pNN20)<br>• Zero-crossings	[85,86]
Stationarity	• Internal Degree of Stationarity<br>• Modified Integral Degree of Stationarity<br>• Modified Mean Degree of Stationarity<br>• Median<br>• Standard Deviation of Mean Vector<br>• Standard Deviation of Standard Deviation Vector	[85]
Entropy	• Fuzzy entropy<br>• Approximate entropy<br>• Sample entropy<br>• Shannon Entropy<br>• Permutation entropy<br>• Slope Entropy<br>• correct conditional entropy<br>• Improved multiscale permutation entropy	[80,87]

	• Multiscale sign series entropy	
Variability	• Interquartile Range<br>• Variability Range<br>• Standard Deviation<br>• Variance<br>• Average amplitude change<br>• Mean Resting Rate<br>• Slope Resting Rate	[80]
Similarity	• Median Coherence<br>• Mutual Information<br>• Modified Integral of Coherence<br>• Mean Coherence<br>• Modified Mean Coherence	[85]
Linearity	• Lag dependence function<br>• Population lag dependence function<br>• Linear fit coefficients<br>• Quadratic fit coefficients<br>• Autoregressive modeling<br>• ARIMA-fit coefficients	[64,85]
Correlation	• Cross-correlation<br>• Auto-correlation<br>• Pearson correlation<br>• Autocovariance	[48]
Plot-based	• Explanation: defined specifically for ECG and each RR interval is plotted as a function of the previous RR interval.<br>• VAI: mean of all the absolute value of angular differences between the lines plotted from the original point to every scatter point and the diagonal line, measuring the angular dispersion of all the points<br>• VLI: standard deviation of all distances of scatter points from the original point, measuring the distance dispersion of all the points<br>• SD1: measuring the width of the ellipse and indicating the short-term variability<br>• Probability density distribution of delta RR intervals:<br>○ Blank pane ratio<br>○ Entropy<br>• Activity (variance of time function)<br>• Poincare plot<br>• Recurrence plot	[3,80,88]
Transformation Techniques	• Temporal Pattern (Shapelet) Extraction<br>• Fast Fourier Transform (FFT)<br>• Short-time fourier transform (STFT)<br>• Waveform decomposition:	[64,71,89,90]

	○ EEG decomposition to 5 levels: (43.4-86.8Hz, 21.7-43.4Hz, 10.8-21.7Hz, 5.4-10.8Hz, 2.7-5.4Hz, 0-2.7Hz)<br>• Dual-tree Complex (Discrete) Wavelet Transform: PPG decomposed into six levels to calculate detail coefficients (high frequency) and approximation coefficients (low frequency).<br>• The Daubechies wavelet of order 4 is the most suitable one for automated epileptic seizure detection.<br>• Continuous wavelet transform: ECG signals can be decomposed up to 5 levels using Daubechies-6 (db6) mother wavelet function.<br>• Principal component analysis<br>• Empirical Mode Decomposition<br>• Recurrence quantification analysis<br>• Singular value decomposition<br>• Dynamic time warping time series distances calculation<br>• Derivatives	
Undefined	• Global volatility Index<br>• Hurst Exponents<br>• Fractal Dimension<br>• Higuchi fractal dimension<br>• Principal components Hjorth parameters<br>• Time Series motif features (Symbolic aggregate approximation): used in the Genetic algorithm<br>• Signal-to-noise ratio<br>• Serial Correlation (TSA)<br>• Self similarity (raw)<br>• Periodicity (raw)<br>• Average Maharaj distance (raw)<br>• Signal-to-noise ratio<br>• Pattern variability<br>• DWT-RWE (Discrete Wavelet Transform and Relative Wavelet Energy)<br>• Relative Wavelet Energy<br>• Average of the first 10 scales of multi-scale entropy<br>• Quadratic sample entropy in TQ intervals (TQEn): specific for ECG or PPG<br>• Lempel-ziv complexity (LZC)<br>• Kolmogorov complexity (KC)	[25,29]

(b)

**Signal Name**	**Features**	**Example**
HRV	Frequency-domain:<br>• Spectral power in the range f ≤ 0.4 Hz (Total PWR)<br>• Very low frequency (VLF): 0.0033 - 0.04 Hz<br>• Low frequency (LF): 0.04 - 0.15 Hz<br>• High frequency (HF): 0.15 - 0.4 Hz	[86,91]

	● Total frequency (TF): <= 0.4 Hz<br>● Ratio between Low Frequency and High Frequency (LF/HF)<br>● Spectral Shannon's entropy (SpectEn)<br>Linear, time-domain:<br>● Average of all normal-to-normal intervals (AVNNI)<br>● Mean normal-to-normal interval (NNI)<br>● Standard deviation of normal-to-normal interval (SDNNI)<br>● Root mean square of successive differences between NNIs (RMSSD)<br>● Number of NNIs > 20/50 ms (NN20/NN50)<br>● Proportion of NN20/NN50 among total number of NNIs (pNN20/pNN50)<br>● Standard deviation of differences between NNIs (SDSD)<br>● Total number of NNIs divided by height of NNI histogram (HRV_TI)<br>● Baseline width of the minimum square difference triangular interpolation of the highest peak of the histogram of NNIs (TINN)<br>● Average of all R peaks amplitude (mean_R)<br>● Standard deviation of all R peaks amplitude (std_R)<br>● Average of all RRIs (mean_RR)<br>● Minimum of all RRIs (min_RR)<br>● Median of all RRIs (median_RR)<br>● Coefficient of variation of RR intervals (CVrr)<br>● Difference between maximum and minimum RR intervals (ΔRRImax)<br>● Normalized absolute deviation (NADev)<br>● Normalized absolute difference (NADiff)<br>Time-frequency:<br>● Standard deviation of Haar 's wavelet (HaarWavSD)<br>Nonlinear, phase space:<br>● Poincare plot standard deviations ratio (SD1/SD2)<br>● Spatial filling index, d = 2, bins = 5 (SFI)<br>● Central tendency measure (CTM)<br>● Sequential trend analysis (STA)<br>● Recurrence plot features,d = 2, lag = 1 (REC, LMean, DET, RecShEn, Lam)<br><br>Nonlinear, fractal:<br>● Detrended fluctuation analysis short-term (< = 11 beats) and mid-term (>11 beats) complexity ($DFA_{\alpha1}$, $DFA_{\alpha2}$)<br>Nonlinear, entropy:<br>● Corrected conditional Shannon's entropy (CCE)<br>● Renyi's entropy (RenyiEn)<br>● Sample entropy (SampEn)<br>● Maximum Sample entropy (MaxSampEn)<br>● Fuzzy approximate entropy (FuzzyApEn1)	

	● Maximum Fuzzy approximate entropy (MaxFuzzyApEn)<br>● Multiscale sample entropy (MultiSampEn)<br>Nonlinear, other:<br>● Lempel-Ziv complexity (LZComp)<br>● Multiscale asymmetry index mean (MultiA$_I$Mean)<br>● Standard deviation of Multiscale asymmetry index (MultiA$_I$StDev)<br>● Allan factor<br>Symbolic dynamics:<br>● Alphabet entropy average (AlphEn)<br>● AlphEn average (AlphEnAver)<br>● Maximum AlphEn (MaxAlphEn)<br>● AlphEn variance (AlphEnVar)<br>● Rate of occurrence of AlphEn (Rate_A - Rate_AA)<br>● alphabet AlphEn averages (AverAlphEn B - AverAlphEn AA)	
EEG	Frequency bands:<br>● Delta (< 5 Hz)<br>● Theta (4–7.99 Hz)<br>● Alpha (8–12.99 Hz)<br>● Beta (13–20 Hz)<br>Waveform decomposition:<br>● 43.4-86.8Hz<br>● 21.7-43.4Hz<br>● 10.8-21.7Hz<br>● 5.4-10.8Hz<br>● 2.7-5.4Hz<br>● 0-2.7Hz	[55]
Photoplethy-smography (PPG)	Entropy measures<br>● ApEn<br>● SaEn<br>● PE<br>● IMPE	[92]
Electro-myography (EMG)	Amplitude<br>● First Higher-Order Mean Absolute Value (HOMAV1)<br>● Normalized 1st Higher-Order Mean Absolute Value (HOMAV1n)<br>● Second Higher-Order Mean Absolute Value (HOMAV2)<br>● Normalized 2nd Higher-Order Mean Absolute Value (HOMAV2n)<br>● Mean Absolute Value (MAV)<br>● Peak to Peak Amplitude (P2P)<br>● Peak Amplitude (PK)<br>● Root Mean Square (RMS)<br>● Mean Relative Time of the Peaks (TMNP)<br>● Mean Relative Time of the Valleys (TMNV)<br>Variability	[85]

	• Interquartile Range (IQR)<br>• Range (R)<br>• Standard Deviation (SD)<br>• Variance (VAR)<br>• Mean Resting Rate (MNRR)<br>• Root Mean Square Successive Interval Differences (RMSSD)<br>• Slope Resting Rate (slopeRR)<br>Stationarity<br>• Interal Degree of Stationarity (IDS)<br>• Median (MD)<br>• Modified Integral Degree of Stationarity (MIDS)<br>• Modified Mean Degree of Stationarity (MMNDS)<br>• Standard Deviation of Mean Vector (SDMN)<br>• Standard Deviation of Standard Deviation Vector (SDSD)<br>Entropy<br>• Approximate Entropy (ADEn)<br>• Fuzzy Entropy (FuzzyEn)<br>• Sample Entropy (SampEn)<br>• Shannon Entropy (ShannonEn)<br>• Spectral Entropy (SpectralEn)<br>Linearity<br>• Lag Dependence Function (LDF)<br>• Population Lag Dependence Function (PLDF)<br>Similarity<br>• Correlation Coefficient (CC)<br>• Median Coherence (MDCOH)<br>• Mutual Information (MI)<br>• Modified Integral of Coherence (MICOH)<br>• Mean Coherence (MNCOH)<br>• Modified Mean Coherence (MMNCOH)<br>Frequency<br>• Bandwidth (BW)<br>• Center Frequency (CF)<br>• Median Frequency (MDF)<br>• Mean Frequency (MNF)<br>• Mode Frequency (MOF)<br>• Zero Crossings (ZC)	
Electro-Corticography (ECOG)	Low pass and Band pass filtering<br>• Count of spikes/poly spikes<br>• Amplitude of spikes/poly spikes<br>• Mean amplitude of epileptic discharge (MAG)	[93]
Electronic Health Records (EHR)	• Date of death is between the ICU admission and discharge time<br>• 25 Phenotype labels	[94]

Feature Selection Methods and Examples

Supplementary Table S5. Summary of feature selection methods and example papers.

Feature Selection Method Name	Feature Selection Method Brief Description	Example Papers
ANOVA	Uses the ANOVA statistical test to rank contribution of variance in the output to the variance in each feature.	[70]
Chi-Squared	Computes the Chi-squared statistic for each feature to test for independence between each feature and the output	[87]
PCA	Exploits the eigenvectors of the covariance matrix to select the subset of features that contributed most to the output	[57,87]
Relief-F	Compares instances within the training set with the most similar (nearest) instance that is labeled the same, and the most similar (nearest) instance that is labeled differently. Uses these "near-hit" and "near-miss" instances to select which features contributed to the comparison/distinction, respectively.	[55]
Mutual-I	Incrementally selects the feature with the highest effect on the joint probability density function between the input and output.	[55]
Fisher-S	Each feature's Fisher-score is calculated by computing the distance between each class's mean of the feature, divided by the variances of the feature in each.	[55]
Minimum Redundancy Maximum Relevance	Incrementally add features to the selected set based on mutual information until the set reaches termination condition. Average mutual information between selected features and its category, and between unselected features and its category are Relevance and Redundancy.	[57,95]
Compensation Distance Evaluation Technique (CDET)	Find the feature sets with the highest difference between two data sets by calculating a value that describes the quality of a feature parameter to separate the condition.	[61]
Temporal interpreted tolerance rough set attribute selection	The proposed attribute selection method determines the attribute relevance and significance based on the temporal pattern mean and trend for each attribute. A temporal similarity measure computes the similarity for each attribute based on its temporal pattern. This temporal based similarity measure defines the lower approximations to construct positive regions. The significance of the attribute is measured using temporal tolerance based degree of dependency. The computed significance attributes are used to predict the effectiveness of the attribute in the classification process.	[56]
Rank-based feature	Descending order of the features' z-value of area under its receiver	[95]

selection according to their relevance with the class labels	operating characteristics curve. Top-ranked features are removed to eliminate their relevance that might be redundant during classification	
Recursive Feature Elimination (RFE) wrapper approach	Searching for a subset of features by fitting the model, ranking features by importance, discarding the least important features, and refitting the mode until a specified number of features remains.	[54]
Dynamic Time Warping	Calculate an optimal distance between two time series with lengths respectively	[79]
Data drift sensitivity estimate	The proposed framework attempts to identify drift sensitive features by generating an artificial dataset containing all rows of the training and validation datasets. All feature importance estimations are then performed by training an ERT classifier with 1000 trees and using its feature importance estimates Likewise, an artificial target label (i.e. class) is generated, which denotes from which dataset the corresponding row originates.	[64]
Diversified Forward-Backward (DFB) (Wrapper method)	It is a wrapper feature selection method, which trains a model with the training set and evaluates it with the validation set, aiming to evaluate the quality of a feature set. It is parallelized and uses a greedy heuristic to narrow down the search space.	[64]
